# Early Tumor Shrinkage and Depth of Response Evaluation in Metastatic Pancreatic Cancer Treated with First Line Chemotherapy: An Observational Retrospective Cohort Study

**DOI:** 10.3390/cancers11070939

**Published:** 2019-07-04

**Authors:** Caterina Vivaldi, Lorenzo Fornaro, Carla Cappelli, Irene Pecora, Silvia Catanese, Francesca Salani, Andrea Cacciato Insilla, Emanuele Kauffmann, Francescamaria Donati, Giulia Pasquini, Valentina Massa, Niccolò Napoli, Monica Lencioni, Piero Boraschi, Daniela Campani, Ugo Boggi, Davide Caramella, Alfredo Falcone, Enrico Vasile

**Affiliations:** 1 Department of Translational Research and New Surgical and Medical Technologies, University of Pisa, Via Savi 6, 56126 Pisa, Italy; 2 Division of Medical Oncology, Pisa University Hospital, Via Roma 67, 56126 Pisa, Italy; 3 Department of Diagnostic Imaging, Pisa University Hospital, Via Paradisa 2, 56124 Pisa, Italy; 4 Department of Surgical, Medical, Molecular Pathology and Critical Area, Division of Surgical Pathology, Pisa University Hospital, Via Paradisa 2, 56124 Pisa, Italy; 5 Department of Transplant and General Surgery, Pisa University Hospital, Via Paradisa 2, 56124 Pisa, Italy; 6 Diagnostic and Interventional Radiology, University of Pisa, Via Paradisa 2, 56124 Pisa, Italy

**Keywords:** early tumor shrinkage, depth of response, pancreatic cancer, FOLFOXIRI, gemcitabine plus nab-paclitaxel

## Abstract

Early tumor shrinkage (ETS) and depth of response (DoR) predict favorable outcomes in metastatic colorectal cancer. We aim to evaluate their prognostic role in metastatic pancreatic cancer (PC) patients treated with first-line modified-FOLFIRINOX (FOLFOXIRI) or Gemcitabine + Nab-paclitaxel (GemNab). Hence, 138 patients were tested for ETS, defined as a ≥20% reduction in the sum of target lesions’ longest diameters (SLD) after 6–8 weeks from baseline, and DoR, i.e., the maximum percentage shrinkage in the SLD from baseline. Association of ETS and DoR with progression-free survival (PFS) and overall survival (OS) was assessed. ETS was reached in 49 patients (39.5% in the FOLFOXIRI, 29.8% in the GemNab group; *p* = 0.280). In the overall population, ETS was significantly associated with better PFS (8.0 vs. 4.8 months, *p* < 0.001) and OS (13.2 vs. 9.7 months, *p* = 0.001). Median DoR was −27.5% (−29.4% with FOLFOXIRI and −21.4% with GemNab, *p* = 0.016): DoR was significantly associated with better PFS (9.0 vs. 6.7 months, *p* < 0.001) and OS (14.3 vs. 11.1 months, *p* = 0.031). Multivariate analysis confirmed both ETS and DoR are independently associated with PFS and OS. In conclusion, our study added evidence on the role of ETS and DoR in the prediction of outcome of PC patients treated with first-line combination chemotherapy.

## 1. Introduction

Pancreatic cancer (PC) is the seventh leading cause of cancer-related death worldwide and is one of the most aggressive tumor types, with almost as many deaths (*n* = 432,000) as incident cases (*n* = 459,000) [1].

Considering all stages, from 2014 to 2018, the overall five-year survival rate increased from 6% to 9%, but it still stands very poor [2]. The majority of patients have metastatic disease at the time of diagnosis and a five-year survival rate of 3% [3].

Although in recent years research has been focusing on molecular alterations in order to develop targeted treatments [4], to date chemotherapy remains the standard of care in advanced disease. In the metastatic setting, two important clinical trials recently changed the standard of care from single-agent gemcitabine to combination chemotherapy [5,6]. Indeed, in the phase III PRODIGE4/ACCORD11 study, Conroy et al. demonstrated that FOLFIRINOX (5-fluorouracil, irinotecan, and oxaliplatin) was associated with a significant improvement in median overall survival (OS), global health status, and quality of life, as compared to Gemcitabine alone [5]. Our group, with a modified schedule of the Gruppo Oncologico Nord Ovest FOLFOXIRI regimen [7], obtained similar results. Likewise, in the MPACT trial, Von Hoff et al. demonstrated that the Gemcitabine plus Nab-paclitaxel (GemNab) regimen was associated with a longer OS in comparison to Gemcitabine monotherapy [5]. Since then, these two regimens (triplet or doublet combination) have been used as gold standard first-line treatments for patients with advanced PC in good clinical conditions [8].

In the last years, the availability of these different treatment options for advanced PC has highlighted the need to identify factors that may predict outcomes and define the quality of response (in terms of precocity and depth) in patients undergoing combination chemotherapies. In addition, no specific data suggest the use of one regimen over the other one, even though they largely differ with regard to route of administration, side effects and costs [9].

While early tumor shrinkage (ETS) and depth of response (DoR) have been convincingly correlated with survival in metastatic colorectal cancer [10,11,12], few data are available for other gastrointestinal malignancies [13] and, in particular, for metastatic PC. The first evidence in this field comes from a limited study including 59 advanced PC patients treated with FOLFIRINOX, suggesting that early response to chemotherapy may predict a favorable outcome [14]. Moreover, an exploratory analysis from MPACT investigators seems to demonstrate a significant tumor shrinkage with GemNab in both primary pancreatic and metastatic lesions [15], leading the way to ongoing evaluation of GemNab in locally advanced disease [16].

Since the available data are still scarce, our aim is to evaluate the prognostic role of ETS and DoR in a larger cohort of metastatic PC patients treated with modern combination regimens such as FOLFOXIRI and GemNab.

## 2. Results

### 2.1. Patients Characteristics and Treatment Efficacy

One hundred thirty-eight patients treated at our institution from August 2010 to November 2017 were enrolled. Eighty-one received FOLFOXIRI, while 57 were treated with GemNab. The most relevant characteristics of the study population are summarized in Table 1.

With a median follow-up of 34.3 months, a median of six cycles of chemotherapy were administered to each patient (range: 1–16). Considering the entire population, median progression-free survival (PFS), post-progression survival (PPS) and OS were 6.2, 5.0 and 10.9 months, respectively. The response rate (RR) was 34% (47 patients, all partial responses), while the disease control rate (DCR) was 71% (98 patients). At the time of analyses, 134 patients (97%) had progressed and 102 (74%) had died. Of 134 patients experiencing disease progression, 98 (73%) received a second-line chemotherapy treatment: of these, 65% were given combination regimens (GemNab, Folfox, Folfiri or Gemcitabine-Capecitabine doublets), while 35% were treated with monotherapies (mainly Gemcitabine). In detail, the more frequently chosen regimens were as follows: GemNab in 26% of cases, Gemcitabine monotherapy in 23%, Folfiri in 23% and Folfox in 9%.

Looking separately at the two cohorts of therapy, the FOLFOXIRI group reached a RR and DCR of 36% and 70%, respectively, while in the GemNab group a RR of 35% and a DCR of 72% were observed. As expected, median PFS (6.4 vs. 6.2 months, *p* = 0.55) and OS (11.5 vs. 10.6 months, *p* = 0.32) did not differ significantly between FOLFOXIRI and GemNab. With respect to subsequent treatments, 67/81 (83%) patients who progressed after first-line FOLFOXIRI received a second-line one. Of these, the vast majority (76%) was administered a Gemcitabine-based regimen, as expected (GemNab, Gemcitabine alone, Gemcitabine-Capecitabine in 36%, 30% and 10% of cases, respectively). Moving to the GemNab population, 31/53 (58%) non-responding patients of the GemNab cohort received a second line chemotherapy. Most of them were fit for combination regimens: indeed, 39% of patients were administered Folfiri and 29% Folfox.

### 2.2. ETS and DoR

In the entire population, at the first radiological evaluation at 6–8 weeks after treatment initiation the median reduction of the sum of target lesions’ diameters (SLD) from baseline was −8.39% and ETS was reached in 49 patients (35.5%) (39.5% in the FOLFOXIRI and 29.8% in the GemNab group, respectively; *p* = 0.280).

Among 102 evaluable patients, the median value of DoR was −27.52% (−29.4% with FOLFOXIRI and −21.4% with GemNab, *p* = 0.016); median time to DoR was 3.15 months (interquartile range 2.5–5.7 months). Similar to ETS, 55.2% of the patients in the FOLFOXIRI group and 43.2% in the GemNab one achieved a DoR equal to or superior than the median value (*p* = 0.230) (Table 2). On the other hand, considering DoR as a continuous variable, patients in the FOLFOXIRI cohort achieved a significantly higher DoR (*p* = 0.037). Moreover, looking at DoR quartile distribution, 20 out of 58 patients in the FOLFOXIRI group and five out of 44 patients in the GemNab group laid in the quartile with the highest DoR (*p* = 0.007). Median time to DoR was 3.9 months (interquartile range 2.5–5.8 months) in FOLFOXIRI cohort and 2.9 months (interquartile range 2.4–5.6 months) in GemNab cohort, respectively (*p* = 0.293).

### 2.3. Survival Analyses

In the whole population, at univariate analyses greater values of both ETS and DoR were significantly associated with better PFS (both *p* < 0.05) and OS (both *p* < 0.05) (Table 3).

At univariate analysis, the other significant factor with respect to PFS was ECOG performance status (PS) 0 (median PFS 7.3 vs. 5.5 months, *p* = 0.005), while ECOG PS 0 (median OS 13.9 vs. 9.5 months, *p* < 0.001) and the absence of liver metastasis (median OS 15.8 vs. 10.1 months, *p* = 0.039) were significantly correlated to OS (Table 3).

#### 2.3.1. ETS: Correlation with Survival

**Figure 1:**
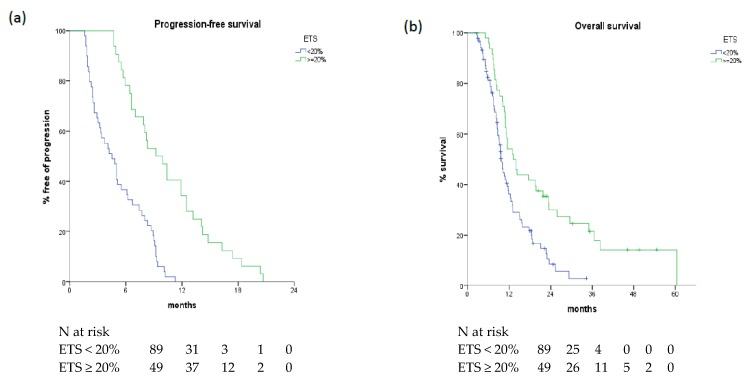
Progression-free survival (**a**) and overall survival (**b**) according to ETS values in overall population.

In the overall population, ETS as a continuous variable was significantly associated with PFS (*p* < 0.001) and OS (*p* < 0.001). Patients achieving ETS ≥ 20% showed a longer PFS than those achieving a tumour shrinkage < 20% (median 8.0 vs. 4.8 months, *p* < 0.005) as well as a longer OS (median 13.2 vs. 9.7 months, *p* = 0.001) (Figure 1).

**Figure 2:**
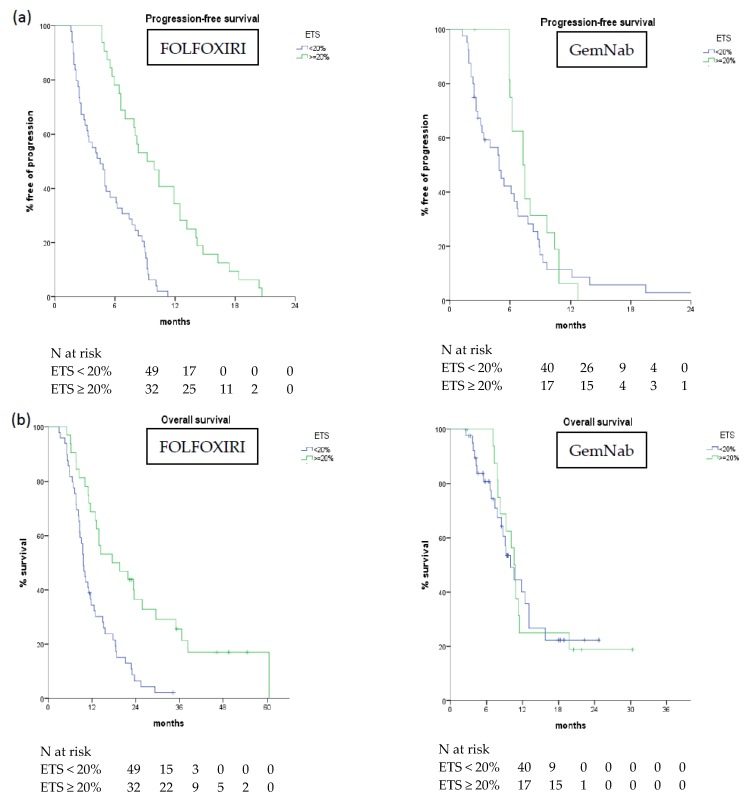
Progression-free survival (**a**) and overall survival (**b**) according to ETS in FOLFOXIRI and GemNab group.

Similar results were observed in the FOLFOXIRI cohort: those who had a SLD reduction ≥20% demonstrated an improved OS (median 17.5 vs. 9.6 months, *p* < 0.001) and PFS (median 9.2 vs. 4.5 months, *p* < 0.001) (Table 4). On the other hand, in the GemNab cohort the presence of ETS ≥ 20% was not associated with OS (median 10.0 vs. 10.6 months, *p* = 0.853) or PFS (median 7.3 vs. 4.9 months, *p* = 0.209) (Table 4, Figure 2).

#### 2.3.2. DoR: Correlation with Survival

**Figure 3:**
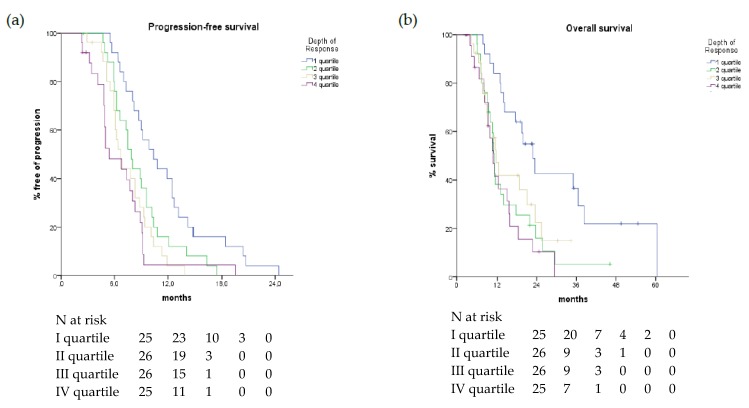
Progression-free Survival (**a**) and Overall Survival (**b**) curves according to DoR quartiles in overall population.

In the overall assessable population, a highly significant association of DoR, both as a continuous and a discrete variable, with PFS and OS was found (Figure 3, Table 3).

DoR was also correlated with improved PPS (HR 1.003, 95% CI 1.003–1.022, *p* = 0.007) and patients achieving deeper responses had better outcome in terms of PPS compared to the others (median 12.9 vs. 3.8 months, *p* = 0.002).

**Figure 4:**
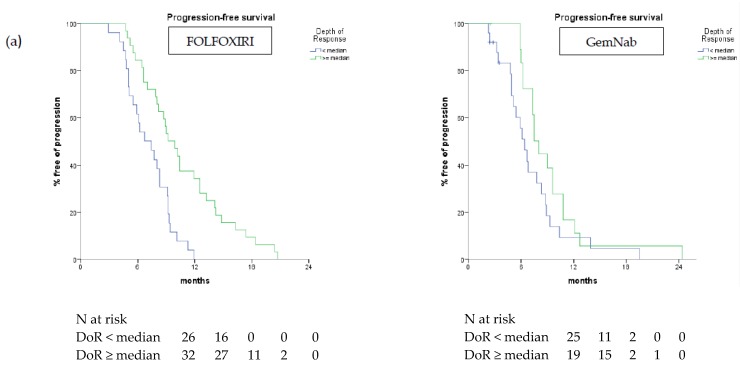

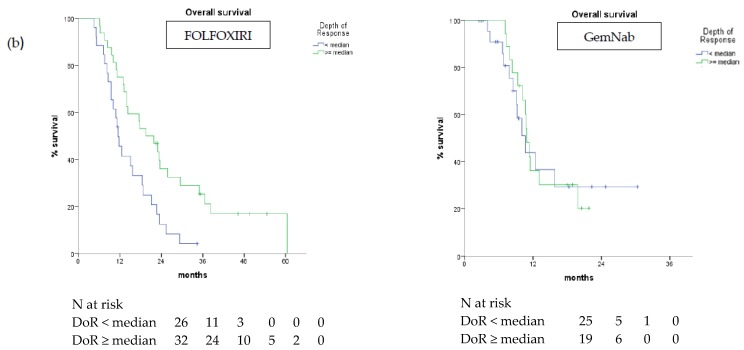
Progression-free survival (**a**) and overall survival (**b**) curves according to DoR ≥ vs. < median value in FOLFOXIRI and GemNab groups.

Considering the two treatment cohorts separately DoR was associated with better PFS and OS when evaluated as a continuous (PFS *p* < 0.001, OS *p* = 0.001) and a discrete variable (PFS *p* < 0.001, OS *p* = 0.005) in patients treated with FOLFOXIRI. In the GemNab group, when evaluated as a continuous variable DoR was associated with better PFS (*p* = 0.001) but not OS (*p* = 0.175), while when considered as a discrete variable it did not reach statistical significance (PFS *p* = 0.093; OS *p* = 0.818). In the FOLFOXIRI group, patients with deeper responses had longer PFS and OS. In particular, median PFS and median OS were 9.9 months and 22.9 months in patients belonging to the first quartile vs. 7.7 months (*p* = 0.004) and 11.7 months (*p* = 0.005), respectively, of the other groups (Figure 4).

Among patients treated with GemNab, a deeper response was significantly associated with PFS (median 10.8 vs. 6.7 months, *p* = 0.031) but the correlation with OS did not formally reach statistical significance (median 19.8 vs. 10.6 months, *p* = 0.097) (Figure 4). All survival data comparisons between the two treatment groups are summarized in Table 4.

#### 2.3.3. Multivariate Analysis

Multivariate analysis confirmed the association of ETS (*p* = 0.033), DoR (*p* < 0.001) and ECOG PS (*p* = 0.004) with PFS. Looking at OS, the independent prognostic role of ETS (*p* = 0.023) and DoR (*p* < 0.0001) was confirmed in multivariate analysis, together with ECOG PS (*p* < 0.0001) and liver involvement (*p* = 0.003) (Table 5).

## 3. Discussion

The present analysis is one of the first aiming to investigate the role of ETS and DoR in patients with metastatic PC. Patients achieving tumor response to chemotherapy are known to have a better prognosis but data regarding the quality of the response in terms of precocity and depth in advanced PC are lacking as well as head-to-head comparison between the two most common first-line chemotherapy regimens used, such as FOLFIRINOX and GemNab.

According to our results, ETS and DoR significantly correlate with survival outcome in patients with advanced PC treated with combination chemotherapy. The association with both PFS and OS is maintained in the multivariate model together with other well-known clinical prognostic variables, underlining how an early and deep tumor shrinkage may represent an independent prognostic factor not only in terms of progression delay, but also in extending survival. To the best of our knowledge, our report is the first that demonstrates the prognostic value of DoR in PC; moreover, we confirmed previously published data for ETS in a larger patient cohort [14]. The prognostic role of PS and liver involvement that emerged from our analysis had already been identified in ACCORD11/PRODIGE4 and MPACT trial analyses [5,17], thus strengthening the value of our results in an independent population treated at a single Institution.

As our aim was to preliminarily evaluate the prognostic role of ETS and DoR in two different parallel cohorts and not to make a comparison between the two regimens in terms of efficacy, no significant difference was noticed in terms of ETS between FOLFOXIRI and GemNab, while results in terms of DoR seem to favor the triplet regimen. The difference between the two regimes in terms of DoR emerges both considering the depth measurements as a continuous variable, as well as looking at patient distribution among the DoR quartiles. Due to the retrospective nature of our work, patients enrolled in the two cohorts may be imbalanced as regarding baseline characteristics and this aspect may have influenced compliance to treatment and activity results. While randomized comparisons are lacking, these data could support the choice of a more intensive regimen such as FOLFIRINOX as a preferable upfront option in those cases where a greater shrinkage is required, such as patients with high tumor burden, symptomatic disease or in the locally advanced setting.

Moving towards the separate evaluation of the two treatment cohorts, we noticed that the prognostic value of ETS was maintained in the FOLFOXIRI cohort while only a trend towards statistical significance was reached in the GemNab cohort. One could argue that this discrepancy could be related to the difference in terms of activity between the two regimens, which could be greater than the difference observed on long-term survival outcomes. Furthermore, the imbalance between the two cohorts in terms of number of patients (the subjects treated with FOLFOXIRI represent 2/3 of the entire population) may have also affected the results. Similar considerations apply to DoR that maintains its prognostic role with FOLFOXIRI for both PFS and OS, while it was significantly correlated only to OS with GemNab.

Some limitations of our study should be acknowledged. It is a retrospective experience conducted in a single center and patients in the two treatment cohorts are not matched by number or by baseline characteristics. However, we observed similar results with FOLFOXIRI and GemNab in our series when compared to those reported in the highly selected populations enrolled in phase III trials and we confirmed the relevance of first-line treatment in determining the survival outcomes in this challenging scenario.

Therefore, validation of our findings in larger, prospective studies is essential in order to definitively validate ETS and DoR as surrogate endpoints in PC. In particular, an ETS ≥ 20% has proved to be a simple, reproducible parameter able to predict outcome in terms of PFS and OS, also showing the advantage of earlier assessment compared to RECIST response in routine practice. Moving from this consideration, it could be interesting to use ETS as a selection or stratification criterion in order to enhance modern strategies in PC treatment, such as sequential treatments with different regimens: nowadays, results achieved with such an approach in unselected populations have been indeed modest or inconclusive [18,19]. On the other hand, the correlation of DoR with PPS makes it a suitable parameter to anticipate the possibility of administering subsequent lines of treatment in metastatic PC patients.

## 4. Materials and Methods

We retrospectively evaluated patients diagnosed with metastatic PC who received first-line combination chemotherapy at our institution between August 2010 and November 2017. All the included patients had a histologically-confirmed diagnosis of pancreatic carcinoma, evidence of distant organ/lymph-nodes involvement or local recurrence not susceptible to loco-regional treatments, good clinical conditions at enrollment (ECOG PS ≤ 1) and underwent a radiological assessment at baseline (performed <28 days from first treatment cycle), after 6–8 weeks since the first drug administration and every eight weeks thereafter until disease progression. Objective responses were evaluated according to RECIST criteria v.1.1.

Each patient received intravenous (i.v.) infusion of oxaliplatin (85 mg/sqm), irinotecan (165 mg/smq), 5-fluorouracil (3200 mg/sqm 48 h continuous infusion) and folinic acid (200 mg/sqm), all administered on day 1 every 14 days in the FOLFOXIRI regimen, or i.v. infusion of gemcitabine (1000 mg/sqm) plus nab-paclitaxel (125 mg/sqm) administered on days 1, 8 and 15 of a 28-day cycle in the GemNab regimen. Treatments were administered until disease progression, unacceptable toxicity or patient’s consent withdrawal. Dose adjustment of single drugs were made as per routine clinical practice and drugs labels.

Two independent investigators evaluated RECIST response, and any controversy was solved by a third independent radiologist with expertise in PC assessment. The independent radiologist was not aware of the regimen received by the patient. ETS and DoR were assessed for each patient as follows: ETS was defined as a reduction ≥20% of the SLD at radiological assessment after 6–8 weeks from treatment start. DoR was defined as the maximum percentage reduction of the SLD, with respect to baseline evaluation, i.e., DoR = [(SLD at best response) − (SLD at baseline) × 100]/(SLD at baseline)%). Patients who presented the onset of new lesions at first radiological assessment were excluded from DoR analyses. ETS was considered both as continuous and binary variable (≥20% vs. <20%), whereas DoR was considered either as continuous, as binary (≥median vs. <median value) and ordinal (with four levels based on quartile distribution) variable. DoR quartiles were defined as follows: I quartile: −81.8% to −44.87%; II quartile: −44.86% to −27.52%; III quartile: −27.51% to −7.34%; IV quartile: −7.33% to +48%.

Clinical, pathological and laboratory data were collected at baseline and throughout treatment duration (see Table 1).

Estimations of time-to-event curves were generated by the Kaplan–Meier method. OS was defined as the time from the first day of treatment until the day of death from any cause. PFS was defined as the time from the first day of treatment until the day of disease progression or death from any cause. PPS was defined as the time between the first documented disease progression and death. Patients alive at the time of analyses were censored at the date of their last follow-up visit, whereas those without disease progression were censored at the time of the last radiologic assessment.

Correlation of ETS and DoR with PFS and OS were co-primary endpoints. We used the log-rank test to compare OS and PFS, setting significance at *p* < 0.05 for a two-sided test. The hazard ratios (HRs) and corresponding 95% confidence intervals (CIs) were calculated by a stratified Cox proportional hazards model. For PFS and OS we initially performed a univariate assessment of the prognostic effect of each explored determinant, then a multivariate analysis was carried out using a stepwise Cox proportional hazards regression modelling and setting statistical significance at *p* < 0.05. The correlation of ETS and DoR with treatment where performed by chi-square test and Mann–Whitney test. Statistical analyses were carried out using statistical software packages SPSS 20.0 (IBM, Chicago, IL, USA) and Graphpad v8.0 (GraphPad Software, San Diego, CA, USA).

## 5. Conclusions

The improvement of therapeutic options for metastatic PC prompted the need for the identification of markers predicting the efficacy of first-line therapy and reliably anticipating patient prognosis, in order to guide clinician’s choice toward the most appropriate treatment for the individual subject.

Our results obtained in a large retrospective cohort of metastatic PC patients treated with standard regimens such as modified FOLFIRINOX and GemNab suggest that an earlier and deeper tumor shrinkage is able to predict long-term outcome of PC patients. Further validation of our findings in independent prospective cohorts is warranted before implementing the use of ETS and DoR in clinical trials and in routine patients’ management.

## Figures and Tables

**Table 1:** Patients’ characteristics at baseline.

Characteristics	All Patients		FOLFOXIRI Cohort		GemNab Cohort	
	*n* = 138	%	*n* = 81	%	*n* = 57	%
Age years, median (range)	64 (41–76)	-	62 (41–75)	-	68 (47–76)	
Age (years)						
<65	80	58	60	74	20	35
≥65	58	42	21	26	37	65
Gender						
Male	71	51.4	40	49.4	31	54.4
Female	67	48.6	41	50.6	26	45.6
ECOG Performance Status						
0	84	68.4	62	76.5	22	38.6
1	54	39.2	19	23.5	35	61.4
Histology: Adenocarcinoma						
Ductal	123	89.1	74	91.4	49	86
On IPMN	12	8.7	6	7.4	6	10.6
Acinar	2	1.4	1	1.2	1	1.7
Squamous	1	0.7	-	-	1	1.7
Site of primitive tumor						
Head/uncinate process	65	47.1	39	48.1	26	45.6
Body/tail	73	52.9	42	51.9	31	54.4
Number of metastatic sites						
Median (range)	2 (1–5)	-	2 (1–5)		2 (1–4)	
Synchronous disease						
Yes	101	73.2	58	71.6	43	75.4
No	37	26.8	23	28.4	14	24.6
Metastatic sites						
Liver	108	78.3	65	80.2	43	75.4
Peritoneum	41	29.7	27	33.3	14	24.6
Lung	32	23.2	14	17.3	18	31.6
Bones	4	2.9	2	2.5	2	3.5
Local recurrence	7	5.1	3	3.7	4	7
Lymph nodes	20	14.4	4	4.8	16	28
Previous treatments						
Curative-intent surgery	37	26.9	24	29.6	13	22.8
Adjuvant chemotherapy	25	18.1	18	22.2	7	12.3
Ca 19.9 [KU/L]						
Normal value	27	19.5	15	18.5	13	22.8
<59 ULN	60	43.4	39	48.1	22	38.6
≥59 ULN	42	30.4	20	24.7	20	35.1
Not evaluable	9	6.5	7	8.6	2	3.5

Abbreviations: ECOG, Eastern Cooperative Oncology Group; Gemcitabine Nab-Paclitaxel (GemNab); IPMN, Intraductal papillary mucinous neoplasm; ULN, Upper Limit of Normal.

**Table 2:** ETS and DoR distribution according to treatment.

ETS and DoR Cut-Offs	All Patients *n* (%)	FOLFOXIRI *n* (%)	GemNab *n* (%)	*p*-Value
ETS				
≥20%	49 (35.5)	32 (39.5)	17 (29.8)	0.28
<20%	89 (64.5)	49 (60.5)	40 (70.2)	
DoR				
I quartile (−81.81%–−44.87%)	25	20 (34.5)	5 (11.3)	0.039
II quartile (−44.87%–−27.52%)	26	12 (20.7)	14 (31.8)	
III quartile (−27.52%–−7.34%)	26	15 (25.8)	11 (25)	
IV quartile (−7.33%–+48%)	25	11 (18.9)	14 (31.8)	
≥median	51	32 (55.2)	19 (43.2)	0.23
<median	51	26 (44.8)	25 (56.8)	

Abbreviations: GemNab, Gemcitabine Nab-Paclitaxel; ETS, early tumour shrinkage; DoR, depth of response.

**Table 3:** Impact of clinical and pathologic features on PFS and OS (univariate analysis) in the whole population.

Clinical and Pathological Features	PFS		OS	
	HR (95% CI)	*p*-Value	HR (95% CI)	*p*-Value
ECOG performance status				
0 vs. 1	1.655 (1.160–2.361)	0.005	2.216 (1.479–3.320)	<0.001
Gender				
male vs. female	1.020 (0.725–1.435)	0.907	1.124 (0.769–1.643)	0.547
Sites of metastases, yes vs. no				
Liver	0.774 (0.513–1.166)	0.22	0.606 (0.375–0.980)	0.041
Lung	1.221 (0.899–1.994)	0.165	1.416 (0.868–2.311)	0.164
Peritoneum	0.974 (0.670–1.417)	0.891	0.942 (0.622–1.425)	0.776
Number of metastatic sites				
1–2 vs. 3–5	0.905 (0.622–1.316)	0.6	1.324 (0.811–1.882)	0.324
Primary site				
head-uncinate process vs. body-tail	0.718 (0.509–1.013)	0.059	0.844 (0.577–1.235)	0.382
Previous surgery on primary tumour				
yes vs. no	1.026 (0.694–1.516)	0.897	1.422 (0.912–2.218)	0.12
Ca19.9 level				
<ULN vs. ≥ULN	0.803 (0.523–1.235)	0.318	1.332 (0.798–2.224)	0.273
ETS				
≥20% vs. <20%	0.444 (0.308–0.640)	<0.001	0.493 (0.324–0.750)	0.001
As a continuous variable	1.029 (1.023–1.035)	<0.001	1.012 (1.008–1.016)	<0.001
DoR				
<median value vs. ≥median value	0.461 (0.304–0.669)	<0.001	0.601 (0.376–0.960)	0.033
As a continuous variable	1.025 (1.016–1.034)	<0.001	1.019 (1.008–1.029)	<0.001

Abbreviations: ECOG, Eastern Cooperative Oncology Group; HR, hazard ratio (95% CI, 95% confidence interval); OS, overall survival; *p*, *p*-value; PFS, progression-free survival; ULN, upper limit of normal; ETS, early tumor shrinkage; DoR, depth of response.

**Table 4:** Median OS and PFS with respect to ETS and DoR in the two treatment groups.

PFS and OS in Treatment Groups	ETS			DoR		
	≥20%	<20%	*p*-Value	≥Median	<Median	*p*-Value
median PFS (months)						
FOLFOXIRI	9.2	4.5	<0.001	9.2	6.7	<0.001
GemNab	7.3	4.9	0.209	7.5	6.4	0.093
median OS (months)						
FOLFOXIRI	17.5	9.6	<0.001	19.5	11.5	0.005
GemNab	10.6	10	0.853	10.9	10.6	0.818

**Table 5:** Multivariate analysis.

Variables	PFS		OS	
	HR (95% CI)	*p*-Value	HR (95% CI)	*p*-Value
ECOG performance status				
1 vs. 0	1.902 (1.235–2.929)	0.004	3.074 (1.843–5.125)	<0.0001
Presence of liver metastases	-	-		
no vs. yes			0.428 (0.244–0.750)	0.003
ETS				
<20% vs. ≥20%	1.890 (1.054–3.389)	0.033	2.349 (1.126–4.897)	0.023
DoR				
continuous variable	1.035 (1.022–1.049)	<0.0001	1.038 (1.022–1.055)	<0.0001

Abbreviations: ECOG, Eastern Cooperative Oncology Group; HR, hazard ratio (95% CI, 95% confidence interval); OS, overall survival; *p*, *p*-value; PFS, progression-free survival; ETS, early tumor shrinkage; DoR, depth of response.
